# Description and Verification of the Fundamental Current Mechanisms in Silicon Carbide Schottky Barrier Diodes

**DOI:** 10.1038/s41598-019-40287-1

**Published:** 2019-03-06

**Authors:** Jordan Nicholls, Sima Dimitrijev, Philip Tanner, Jisheng Han

**Affiliations:** 1 Queensland Micro- and Nanotechnology Centre, Griffith University, Queensland, 4111 Australia; 2 School of Engineering and Built Environment, Griffith University, Queensland, 4111 Australia

## Abstract

Attempts to model the current through Schottky barrier diodes using the two fundamental mechanisms of thermionic emission and tunnelling are adversely impacted by defects and second order effects. This has led to the publication of countless different models to account for these effects, including some with non-physical parameters. Recently, we have developed silicon carbide Schottky barrier diodes that do not suffer from second order effects, such as excessive leakage, carrier generation and recombination, and non-uniform barrier height. In this paper, we derive the foundational current equations to establish clear links between the fundamental current mechanisms and the governing parameters. Comparing these equations with measured current–voltage characteristics, we show that the fundamental equations for tunnelling and thermionic emission can accurately model 4H silicon carbide Schottky barrier diodes over a large temperature and voltage range. Based on the obtained results, we discuss implications and misconceptions regarding barrier inhomogeneity, barrier height measurement, and reverse-bias temperature dependencies.

## Introduction

Work on modelling the carrier transport through a metal–semiconductor contact began 80 years ago, shortly after Schottky’s paper demonstrated the formation of a potential energy barrier at the interface^1^. Thermionic emission theory, first derived by Bethe in 1942^2^, has been shown to be the primary mechanism for the forward-bias current of Schottky barrier diodes manufactured on many different semiconductors. Regarding the reverse-bias current, it has been recognized that a model for the additional tunnelling mechanism has to be added to the thermionic emission, but no unique model could be established that would agree with measurements over any substantial temperature or voltage range. Instead, numerous different models of the tunnelling contribution appear in literature^3–6^, creating a confusion about the responsible physical parameters.

The problem has been the impact of defects and other second order effects, which add highly-variable components to both the forward and reverse currents. For instance, the reverse bias current of silicon Schottky barrier diodes is dominated by carrier generation due to defects with mid-gap energy levels, located in the space charge region^7,8^. Field enhancement at the metal edge has also resulted in increased tunnelling currents for devices without an adequate edge termination^9^. The forward bias response also deviates from the fundamental theory; in particular, a fitting parameter called the ideality factor has been introduced and then attributed to several different phenomena^3,10–12^.

Many authors have developed and applied models for either forward or reverse bias conduction, attempting to account for these additional effects. However, few studies address both bias polarities simultaneously. The separate treatment of the two polarities has resulted in models with questionable physical meaning of their parameters, because their independent fitting results in different values for the two different current directions. One would expect that the metal–semiconductor interface should be able to be described by a single set of fundamental parameters regardless of the direction of the current. While this makes logical sense, it is often ignored in favour of fitting arbitrary parameters to the experimental data. Consequently, this has caused confusion about the values of the physical parameters that describe the interface.

Some groups have attempted a more thorough analysis of the carrier transport. Silicon Schottky barrier diodes with aluminium, copper, silver, and gold anodes have been studied for both current directions^7–9^. The reverse leakage was found to be primarily due to generation^7,8^ or edge leakage^9^. Padovani and Stratton showed that gold–n-type gallium arsenide diodes were dominated by tunnelling in forward and reverse bias, but there were discrepancies in the effective masses and barrier heights between the two bias polarities^3^. Hashizume, Kotani and Hasegawa modelled gallium nitride diodes under both forward and reverse bias over a small voltage range using a thin surface barrier model of the potential energy^13^. Two teams (Okino *et al*.^14^ and Blasciuc-Dimitriu *et al*.^15^) used parameters derived from the forward characteristic to describe the reverse leakage currents observed for silicon carbide (SiC) Schottky barrier diodes.

SiC is a next generation semiconductor suitable for high-voltage and high-temperature power applications. It allows for measurement over a greater range of voltages and temperatures than is possible for traditional silicon devices and, owning to its wide energy gap (3.26 eV for the 4H polytype^16^), additional currents due to generation and recombination are not present in metal–SiC contacts. With an adequate edge termination to eliminate the impact of edge leakage, the metal–SiC interface is the ideal structure for experimental verification of the fundamental equations for the two principle current mechanisms (thermionic emission and tunnelling). SiC Schottky diodes have been commercially available for more than a decade, but these diodes typically use a “junction barrier Schottky” structure with P–N junction pockets at the metal–SiC interface that significantly impact the reverse-bias current. Recently, we have been able to develop “pure” SiC Schottky diodes with an edge termination that removes the perimeter leakage.

In this paper, we show that experimental measurements of our Schottky barrier diodes in both forward and reverse bias can be accurately modelled by the two fundamental current mechanisms (thermionic emission and tunnelling) with a single set of physically meaningful parameters. The model used is derived from fundamental physics, keeping it as simple as possible while still maintaining the good match to experimental data. This work demonstrates that it is possible to manufacture diodes that are free from defects which dominate the current transport.

## The Elements of The Fundamental Physical Model

The fundamental current equations can be derived by counting the number of electrons that arrive at the interface at various energy levels, and determining their contribution to the current. This method is foundational to almost all current transport models for Schottky diodes, and has been described in the past^5,17^. To avoid possible confusion resulting from the proliferation of the number of models while we discuss the key physical parameters in this paper, we will show explicitly the essential modelling elements that are not obvious from the condensed form of the fundamental equation.

To begin, we need to count the number of electrons that hit the interface. Consider all electrons with a given velocity in the *x* direction (normal to the interface) due to thermal motion. Per scattering interval, all electrons within the scattering length will reach the interface. Multiplying the scattering length by the electron concentration (*n*), and using the relationship between the scattering length and the thermal velocity, the number of hits per unit time and area (*N*_*hits*_) is

1 $${N}_{hits}={v}_{th-x}n$$

where *v*_*th−x*_ is the component of the electron velocity normal to the interface. The energy barrier at the interface prevents most of these electrons from contribution to the current. Multiplying by the probability (*P*) that an electron will traverse the interface, either by thermionic emission over the barrier or by tunnelling through the barrier, we arrive at following basic equation for the current density (*j*):

2 $$j=qP{N}_{hits}$$

where *q* is the elementary charge.

Both the probability and the number of hits will vary with the kinetic energy of the electrons. To account for this, we can write eq. (2) in the following differential form, and then integrate over all energy levels to arrive at the total current:

3 $$dj=qP({E}_{kin-x}){N}_{hits}({E}_{kin-x})d{E}_{kin-x}$$

where ${E}_{kin-x}=\frac{{m}^{\ast }{{v}_{th-x}}^{2}}{2}=\frac{{m}^{\ast }{({v}_{th}\cos \theta )}^{2}}{2}$ is the energy associated with motion in the *x* direction, *θ* is the angle between the *x* axis and the direction in which any particular electron approaches the interface, and m* is the effective mass of the electrons. The total thermal velocity of each particular electron is *v*_*th*_.

If we apply the free electron model, then it is possible to derive an explicit form for *N*_*hits*_*(E*_*kin−x*_). The result of this derivation, which is shown in the supplementary materials, is

4 $${N}_{hits}({E}_{kin-x})=\frac{4\pi {m}^{\ast }kT}{{h}^{3}}\,\mathrm{ln}[1+\exp (\frac{{E}_{F}-{E}_{kin-x}}{kT})]$$

where *k* is Boltzmann’s constant, *T* is the absolute temperature, *h* is Plank’s constant, and *E*_*F*_ is the Fermi level. Substituting eq. (4) into eq. (3), we find that

5 $$j=\frac{4\pi q{m}^{\ast }kT}{{h}^{3}}{\int }_{0}^{\infty }P({E}_{kin-x})\mathrm{ln}[1+\exp (\frac{{E}_{F}-{E}_{kin-x}}{kT})]d{E}_{kin-x}.$$

**Figure 1:**
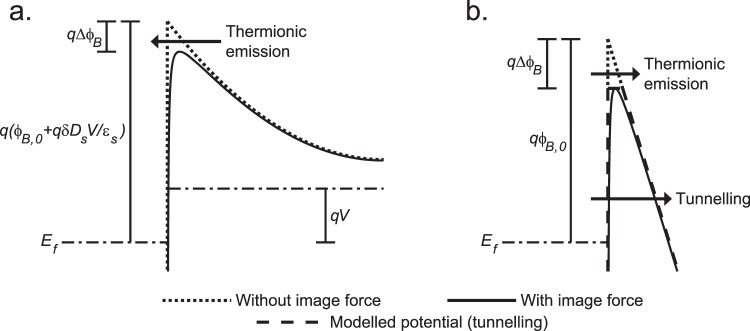
Energy-band diagrams for (**a**) forward and (**b**) reverse bias conduction. The fundamental conduction mechanisms of thermionic emission and tunnelling (for reverse bias) are indicated by the arrows. The dotted lines indicate the shape of the potential energy in the absence of the image force, while the solid lines are the actual potential. In (**b**), the trapezoidal approximation used in eq. (10) is indicated by the dashed line.

This is the foundation of most models of Schottky diode current for both bias polarities. For forward bias (Fig. 1a), we can assume that the quasi-Fermi level is flat throughout the space charge region, and we can neglect tunnelling. Under these assumptions, the probability *P* can be taken as 1 when *E*_*kin−x*_ ≥ *E*_*F*_ + *qϕ*_*B*_ − *qV* and 0 otherwise, where *qϕ*_*B*_ is the energy-barrier height, and *V* is the applied forward-bias voltage. Solving the integral in eq. (5) with these assumptions, we arrive at the well-known thermionic-emission equation for the current from semiconductor to metal only^2^,

6 $$j={A}^{\ast }{T}^{2}\exp (\frac{-q{\varphi }_{B}}{kT})\exp (\frac{qV}{kT})$$

where ${A}^{\ast }=\frac{4\pi q{m}^{\ast }{k}^{2}}{{h}^{3}}$ is the Richardson constant.

A number of factors contribute to the barrier height. To a first order approximation, the barrier height is controlled by the metal work function and the semiconductor electron affinity. However, this alone does not account for the measured barrier heights of many diodes—interface charge and the image force are both important factors that cannot be avoided. The total equation for the barrier height is^18^

7 $$q{\varphi }_{B}=q{\varphi }_{B,0}-q{\Delta }{\varphi }_{B}+\frac{{q}^{2}\delta {D}_{s}}{{\varepsilon }_{s}}V$$

where *qϕ*_*B*,0_ is the zero bias barrier height (excluding the image force), *ε*_*s*_ is the static permittivity of the semiconductor, *δ* is the distance of the interface traps from the metal surface, and *D*_*s*_ is the density of interface traps (units: m^−2^eV^−1^). For the traps, we have assumed a constant density of acceptor type traps whose occupancy follows the applied forward bias. The image force correction (*qΔϕ*_*B*_) is given by $q{\Delta }{\varphi }_{B}=2q\sqrt{\frac{q\xi }{16\pi {\varepsilon ^{\prime} }_{s}}}\,$^18^, where ${\varepsilon ^{\prime} }_{s}$ is the image force permittivity and *ξ* is the surface electric field in the semiconductor (which can be calculated using the one-dimensional Poisson equation). The image force permittivity may be the low or high frequency permittivity, depending on a number of material factors (for instance, does the material fully polarise in response to a carrier’s electric field within the short crossing time^11^). For the set purpose in this paper, we will use the low frequency value, as others have done for SiC^14^.

For reverse bias, the final term in eq. (7) is not included (as the population of traps is controlled by the metal Fermi level in reverse bias), and so the barrier height only varies via the image force. However, now tunnelling can no longer be neglected. Many different models of the probability function have been used^3–6^ and, together with analytical approximations to eq. (5) ^3–5^, has been another contributing factor to the large number of different models.

In this work, we will use a Wentzel–Kramers–Brillouin (WKB) approximation to calculate the probability function for tunnelling. To simplify the fitting, we can approximate the potential energy as trapezoidal, defined by a constant surface electric field, but truncated by the image force. This can be seen as the dashed line in Fig. 1b. In the region where tunnelling occurs, this potential shape is equivalent to the more realistic truncated parabolic potential for high reverse biases (greater than 100 V), because the field near the interface is approximately constant. A non-truncated potential (one where the image-force rounding is included) is not suitable for the type of fitting we are using due to the increase in computation it requires.

The total probability function for reverse bias is given by

8 $$P=\{\begin{array}{cc}1, & {E}_{kin-x}\ge {E}_{F}+q{\varphi }_{B,0}-q{\Delta }{\varphi }_{B}\\ \exp [-\frac{4\pi \sqrt{2{{m}_{t}}^{\ast }}}{hq\xi }\sqrt{{E}_{\varphi }}(\frac{2}{3}{E}_{\varphi }+q{\Delta }{\varphi }_{B})], & {E}_{kin-x} < {E}_{F}+q{\varphi }_{B,0}-q{\Delta }{\varphi }_{B}\end{array}$$

where *m*_t_* is the tunnelling effective mass and *E*_*ϕ*_ = *E*_*F*_ + *qϕ*_*B*,0_ − *qΔϕ*_*B*_ − *E*_*kin−x*_. Note that the tunnelling effective mass is not the same as the effective mass in eq. (6) and will be measured independently. More complex theoretical calculations indicate that *m*_t_* can have an energy dependence; however, our experimental results did not indicate that it was necessary to introduce a more complex parameter than the constant *m*_t_*.

An important consideration is what effective mass value (or Richardson constant) to use in eqs. (5) and (6). The bulk effective masses of the two materials may not be applicable to the interface, as device processing may cause damage or introduce impurities, causing it to no longer behave like bulk metal or semiconductor. In addition, the equations we have outlined here do not account for quantum mechanical reflections or back scattering—these omissions will result in a reduction in the effective mass measured from Schottky diode current–voltage characteristics as compared to theoretical values^19^.

There is also the issue that, in order for the net current to be zero at equilibrium, the conduction in both directions must be governed by the same effective mass. This is despite the fact that these are two very different materials which will not have the same mass, and so there must be some mechanism to equalise the current. Some theoretical treatments suggest that, to a first approximation, the effective mass of the semiconductor should be used^20^. This is because the image-force reduced barrier is entirely located inside the semiconductor, but since the current still originates from the metal its distribution should still be controlled by the metal mass. A more complex analysis involves considering the conservation of momentum parallel to the interface^14^. Doing so results in a shift in *E*_*kin−x*_ based on the energy and mass tensor components parallel to the interface; this can reduce the net current, but in general this alone will not balance the currents.

We propose the following solution to this problem: If we reframe the equilibrium condition from equating the Fermi levels to equating the electron flux, then it is clear that the net current will change the semiconductor depletion width (similar to when a bias is applied) until the forward and reverse currents are balanced. In the case of non-equal effective masses, this means that there will be a shift in the semiconductor quasi-Fermi level with respect to the metal Fermi level, which changes the barrier to electrons on the semiconductor side of the interface. The change in energy barrier (which we will label as *qV*_*m*_) that will balance the two opposing currents is

9 $$q{V}_{m}=kT\,\mathrm{ln}(\frac{{{m}_{m}}^{\ast }}{{{m}_{s}}^{\ast }})$$

where *m*_m_* and *m*_s_* are the effective masses of the metal and semiconductor. This factor should be subtracted from the barrier height in the equation for the semiconductor to metal current. By including it in eq. (6), the current from semiconductor to metal becomes

10 $$j=\frac{4\pi q{{m}_{s}}^{\ast }{k}^{2}{T}^{2}}{{h}^{3}}\exp (\frac{-q{\varphi }_{B}}{kT})\exp (\frac{qV}{kT})\exp (\frac{q{V}_{m}}{kT})=\frac{4\pi q{{m}_{m}}^{\ast }{k}^{2}{T}^{2}}{{h}^{3}}\exp (\frac{-q{\varphi }_{B}}{kT})\exp (\frac{qV}{kT})$$

The result in this new formulation that the effective mass of electrons in the metal determines the current from the semiconductor is also in agreement with experimental results which show that the effective mass is affected by the choice of metal^14,21^.

### Experimental verification

We can see from this derivation that a Schottky barrier diode governed only by the fundamental mechanisms of thermionic emission and tunnelling and with no other second order effects has four parameters which need to be extracted from the current-voltage characteristics in order to fully describe the carrier transport. These are the barrier height (*qϕ*_*B*,0_), the interface trap parameters (*δD*_*S*_), and the two effective masses (*m*_t_* and m*).

**Figure 2:**
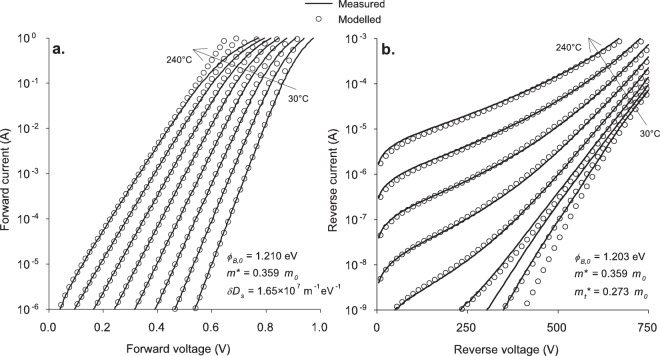
Comparison of measured and modelled current–voltage characteristics of 650-V diodes for both forward (**a**) and reverse (**b**) bias at temperatures from 30 °C to 240 °C in 30 °C steps.

**Figure 3:**
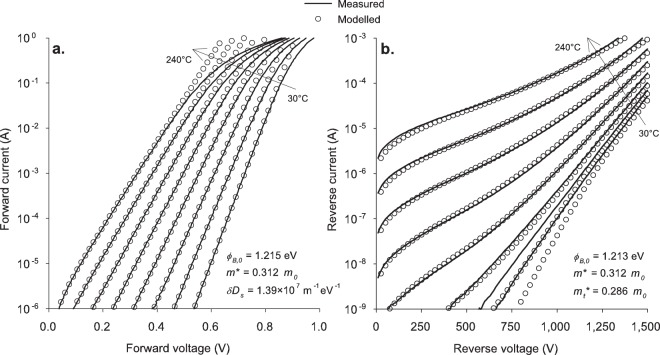
Comparison of measured and modelled current–voltage characteristics of 1200-V diodes for both forward (**a**) and reverse (**b**) bias at temperatures from 30 °C to 240 °C in 30 °C steps.

To verify the fundamental model, we applied it to 4H-SiC Schottky diodes with two different doping profiles. This is in order to determine if they can be described by these four parameters only or if second order effects are necessary to consider. Details of the two doping profiles, obtained from capacitance measurements, are shown in Table 1. The resulting fits of the fundamental equations are plotted against the measured data in Figs. 2 and 3, and the parameters extracted from the fitting are listed in Table 2.

**Table 1:** Doping and thickness of the n-epitaxial layers, obtained by high reverse capacitance–voltage measurements at room temperature.

		Blocking voltage	
		650 V	1200 V
Doping (cm^−3^)	Epitaxial layer	8.5 × 10^15^	5.5 × 10^15^
	Buffer layer	5.4 × 10^17^	1.2 × 10^17^
Epitaxial layer thickness (μm)		4.4	9.5

**Table 2:** Values of the fitting parameters for both diode samples.

		Blocking voltage	
		650 V	1200 V
*ϕ*_*B*,*0*_ (eV)	From forward bias	1.210	1.215
	From reverse bias	1.203	1.213
m*/*m*_*0*_		0.359	0.312
*m*_t_*/*m*_*0*_		0.273	0.286
*δD*_*s*_ (m^−1^eV^−1^)		1.65 × 10^7^	1.39 × 10^7^

The agreement between the model and the experimental data is extremely good for both bias directions on both samples, although there are some areas where the model deviates from the experimental data. In forward bias, the measurements at high currents curve away from the theoretical model. This is due to the series resistance of the diodes, which has not been included here because this effect is well known and its modelling is by straightforward application of Ohm’s law.

**Figure 4:**
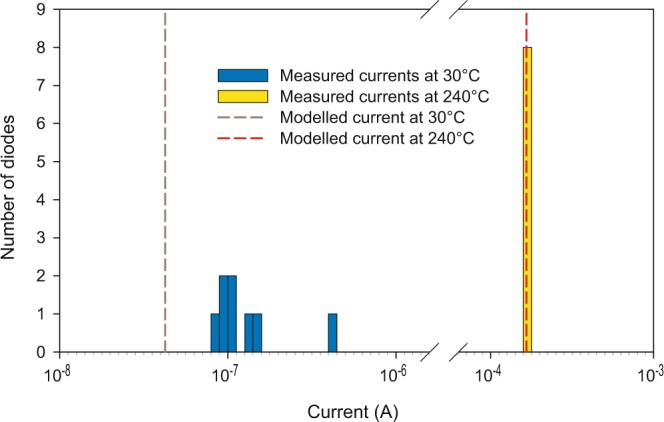
Histogram of the measured currents of eight 1200-V diodes at 1000 V reverse bias. Measurements at 30 °C and 240 °C are shown to compare the relative scatter. The modelled currents at these temperatures and voltage are included as dashed lines.

The fundamental model also fails to describe the reverse bias currents at low temperatures, and in particular low biases, for both diodes. We conclude that this is due to defects, based on an analysis of the variations between diodes on the same wafer (illustrative results are shown in Fig. 4 for the case of 1200 V diodes). At low temperatures, there is considerable scatter in the leakage currents; however, these same diodes have nearly identical currents when measured at high temperatures. This shows that the deviation is due to defects—the model works well at high temperatures, since the current through the main diode area dominates the defect current, but at low temperatures the defect current results in a failure of the model to match the experimental data. This defect current is due to tunnelling and not thermionic emission, since there is no evidence of these defects in the forward characteristics (the forward currents of all of the diodes in Fig. 4 are practically identical). Some possible sources of this defect current are roughness in the main diode area or leakage through the edge termination.

There is also a small difference in the barrier heights between the forward and reverse characteristics. There are two factors which could contribute to this deviation. The first is the use of a trapezoidal approximation of the potential energy, which could cause an error in the fitted barrier height in reverse bias. The second is a small change in the occupation of the interface traps in reverse bias, for instance due to a slight decrease in the quasi-Fermi level in reverse bias^22^. In any case, the agreement between the barrier heights here is very good and is much better than what is typically observed between different barrier height measurement techniques.

### Implications

#### Barrier inhomogeneity

For forward bias conduction, changes in the barrier height with voltage are often modelled with a fitting parameter called the “ideality factor”. In this work, we have successfully accounted for this ideality factor with the image force and interface traps. However, this is not the only possible explanation. In particular, barrier height inhomogeneity has been used to explain the ideality factors of many different diodes. There are several different ways to model inhomogeneity, but the approach that could potentially explain these results without invoking arbitrary non-physical parameters is the Tung model^10^.

The Tung model describes the behaviour of nano-scale patches of lower barrier height amongst a higher barrier background^10^. In particular, he finds that the potential in front of the patches is “pinched off”, which causes the barrier to be voltage dependent^10^. However, we can rule out this type of inhomogeneity for a few reasons. For one, his model predicts temperature dependencies that are not consistent with our measurements, and we find that our data is consistent with the presence of the image force in forward bias, which is in conflict with his model. The biggest argument against that model though is that the low barrier patches should not be pinched off in reverse bias and the current should be dominated by these low barrier patches, but our results show a good agreement between the barrier heights in forward and reverse using the same effective mass and area. This indicates no barrier height inhomogeneity, and therefore we conclude that the Tung model is not suitable for the Schottky diodes used in this paper.

Ideality factors better than 1.2 (with 1 being ideal) are commonly assigned to inhomogeneity (at least for SiC)^23^. The diodes in this paper have ideality factors better than 1.1 but, as explained previously, there is no evidence of barrier height inhomogeneity. For this reason, we would like to caution against the use of barrier height inhomogeneity to explain the ideality factors of diodes without first considering if it is consistent with the reverse characteristic.

#### Barrier height measurement

It has often been observed that different barrier-height measurement techniques obtain different results. Capacitance methods are in general considered more reliable than current and current–temperature methods. A common argument against the use of current methods is that they are heavily impacted by defects in the diode area. Tunnelling, barrier inhomogeneity, and the image force [if it is not included as it is in eq. (7)] can all result in incorrect extraction of the barrier height from current methods. Furthermore, traditional methods of extraction by fitting straight lines to the linear portions of the forward current–voltage characteristics are prone to error. It is for these reasons that researchers have favoured the capacitance method for barrier height measurement. However, capacitance measurements are indirect and require knowledge of the difference between the metal Fermi level and the bottom of the conduction band. As we have shown, the shift due to the different effective masses on either side of the interface will change the relationship between the metal Fermi level and the bottom of the semiconductor conduction band (at equilibrium), causing this method to be in error.

In this paper, we have demonstrated an excellent agreement between the barrier heights extracted using independent (except for the effective mass) fitting between the forward and reverse bias directions. The corroboration of two different measurements gives increased confidence that this barrier height is correct. Additionally, these methods are direct, and all parameters involved in the calculation of the barrier height, except for the semiconductor permittivity, are obtained from measurement of the diodes in question. The present results would suggest that, assuming that the absence of defects has been verified over some temperature range, fitting of the forward and reverse current–voltage characteristics is the most accurate method for barrier height extraction.

#### Temperature dependence of reverse bias current

The strength of the temperature dependence in reverse bias may be surprising, since it should be dominated by the temperature-independent tunnelling probability. Equation (6) does involve a temperature dependent term, but the responsible physical mechanism is not obvious from that summary equation.

**Figure 5:**
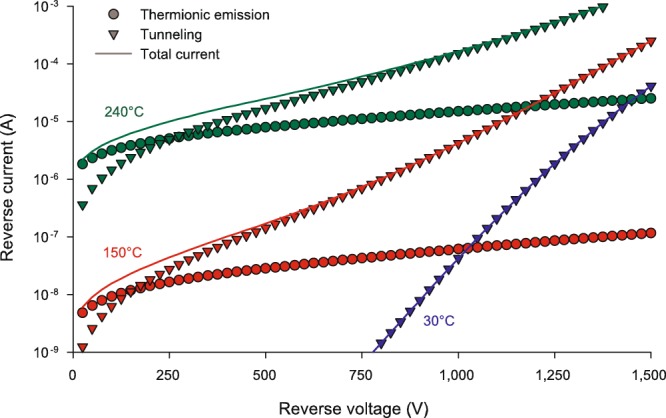
Modelled reverse-bias currents at 30, 150, and 240 °C for the 1200-V diodes. Currents are divided into the two mechanisms: thermionic emission (circles) and tunnelling (triangles). Solid lines indicate the total current, which is the sum of the thermionic and tunnelling components.

To analyse the temperature dependence, let us first divide the modelled reverse current at three temperatures (30, 150 and 240 °C) into the two fundamental mechanisms (thermionic emission and tunnelling). This is shown in Fig. 5. Not only do we find that there is a temperature dependence in the tunnelling current, but it also shows that thermionic emission over the barrier is a significant contributor to the current at higher temperatures. Even this is somewhat surprising considering the height of the barrier, which is greater than 1 eV at all applied biases. Common thinking regarding the distribution of electrons is to consider that the number of electrons with energies greater than 2–3 times the thermal voltage (*kT*/*q*) above the Fermi level is negligible, which implies that there should be no thermionic emission at the temperatures used in this paper.

We can calculate just how many electrons are creating this thermionic current. For instance, at 0 V and 240 °C on the 1200 V sample, the barrier height is 1.19 eV. From this, we calculate that the concentration of electrons in the metal with kinetic energies greater than the height of the barrier is 5.9 × 10^8^ cm^−3^ (using *E*_*F*_ = 13.47 eV^24^). The concentration of free electrons in titanium is about 5.7 × 10^22^ cm^−3^, and so we can see that only one in roughly every 10^14^ electrons contribute to the measured thermionic current. The following analysis can help us visualize this apparently insignificant fraction of electrons. The area of the diodes on the 1200 V sample is 4.08 mm^2^, which means that the number of electrons with energies greater than the barrier height per unit length away from the interface is 2.4 nm^−1^. Since there is no field, and hence no preferred direction of motion in the metal, only half of these will be moving towards the interface. Furthermore, only those within the scattering length can contribute during each scattering time interval. The mean free path in elemental metals range from 1–50 nm^25^; taking *l*_*sc*_ = 10 nm, then there would only be 12 electrons hitting the interface during the scattering time. Finally, some of these electrons will be reflected (either quantum-mechanically or due to a steep incidence angle), so we can expect that fewer than 10 electrons are responsible for the approximately 0.8 μA of electric current flowing from the metal into the semiconductor at 0 V.

The reason that so few electrons can cause such a large current is that the scattering time is extremely short, and that is because the thermal velocity of the electrons is very large. The slowest electrons that can contribute to thermionic emission have kinetic energy equal to the barrier height, and their thermal velocity is 3.6 × 10^6^ m/s, or 1.2% the speed of light. Referring to eqs. (1) and (2), we can see that the large thermal velocities of the electrons with energies higher than the barrier height balances their extremely low concentration, resulting in number of hits corresponding to the observable current of 0.8 μA. It has been known for some time that electrons in metals can have very high velocities, but this fact has eluded the common intuitions and heuristics about Schottky diode current. A possible reason is that textbooks present average values of the thermal velocity, which is well below the velocities of the small number of high-energetic electrons that make the measured current.

With this in mind, we can now explain the temperature dependence of the tunnelling current. As the temperature is increased, the number of electrons at higher energies is also increased. Although the concentration of these electrons is still quite small, their thermal velocity is very large, resulting in a significant increase in the number of hits per unit time, as can be deduced from eq. (1). In addition, at higher energies the tunnelling probability is greater. These two factors result in the tunnelling current increasing with temperature, despite the fact that the tunnelling itself is not temperature dependant. In the past, authors have distinguished this type of current from tunnelling of electrons near the Fermi level (so called “field emission”) with the name “thermionic-field emission”, to describe that it is the tunnelling of electrons that have been thermally excited (but not enough to go over the barrier). We have not made this distinction in this paper, since the boundaries between them are arbitrary.

#### Two dimensional materials

The derivation of metal–semiconductor current transport that is presented in this paper also has implications for the emerging fields of two-dimensional (2D) electronic devices. Some examples of these materials include graphene and the 2D electron gas in GaN/AlGaN heterojunctions. The model presented here assumes a three-dimensional (3D) free electron gas in the semiconductor and the derivation makes it clear that the current equations would be very different for 2D materials. Nonetheless, the particle-wise approach shows that the current from metal to the 2D electron gas, either at the GaN/AlGaN heterojunction or graphene, may be described by the 3D model, whereas a model for the 2D electron gas will be needed for the opposite current. Some groups have attempted to account for these effects and modify the current equations^26^, but there is still more work to be done in this regard.

## Methods

### Device fabrication

The Schottky diodes were fabricated using 100-mm 4H-SiC wafers with (1000) orientation and with commercially grown n-epitaxial layer on the silicon face. Two different doping levels with two different thicknesses were specified, according to the standard design for blocking voltages of 650 V and 1200 V. The measured doping and thickness values are shown in Table 1. Following the preparation of adequate edge-termination rings, titanium and aluminium were deposited and annealed to create the Schottky contact on the n-epitaxial layer. The processing was completed by a standard nickel-silicide contact and silver metallization on the back of the wafer, and a standard polyimide passivation of the top surface.

### Measurement and analysis

Forward current–voltage, reverse current–voltage, and capacitance–voltage measurements were taken at eight different temperatures (from 30 °C to 240 °C in 30 °C increments). All measurements were performed by an Agilent Technologies B1505A Power Device Analyser, connected to an MDC probe station with chuck heating by a Quiet Chuck DC Controller. To measure the doping profile, high voltage capacitance–voltage measurements were taken at room temperature.

Brute-force searches were used to determine the best values of the fitting parameters *qϕ*_*B*,0_, m*, *m*_t_*, and the $\frac{{q}^{2}\delta {D}_{s}}{{\varepsilon }_{s}}$ parameter in eq. (9). The fitting error was calculated using a least squared approach applied to the log current data. For forward bias, all of the eight temperatures were simultaneously used in the search. For reverse bias, the highest temperature was used and additional temperatures were added until the fit was no longer good (indicating defects). This occurred at 90 °C, so the fitting in this paper used only the temperatures greater than or equal to 120 °C. The fitted value of m* from forward bias was used in the reverse bias fitting. Forward bias fitting was limited to currents less than 5 mA, while reverse fitting bias was limited to voltages greater than 100 V. The value of *ε*_*s*_ and ${\varepsilon ^{\prime} }_{s}$ was 9.66*ε*_*0*_^14^, where *ε*_*0*_ is the permittivity of free space.

## Data Availability

The datasets analysed during the current study are available from the corresponding author upon reasonable request.

## Supplementary information

Supplementary Material
