# Supplementary material for: Description and Verification of the Fundamental Current Mechanisms in Silicon Carbide Schottky Barrier Diodes
Source: Sci Rep. 2019 Mar 6;9:3754. doi: 10.1038/s41598-019-40287-1 (PMC6403215; doi:10.1038/s41598-019-40287-1)
Supplement: Supplementary file 1 — Supplementary Material [file 41598_2019_40287_MOESM1_ESM.pdf]

Description and Verification of the Fundamental Current Mechanisms in Silicon Carbide Schottky Barrier Diodes

Jordan Nicholls, Sima Dimitrijev, Philip Tanner, Jisheng Han

Note: Consult the main text for the meaning of any symbols that are not explained here.

First, let us find the concentration of electrons as a function of the total kinetic energy, $E_{kin\text{-}T}$. The number of electrons with kinetic energy between $E_{kin\text{-}T}$ and $E_{kin\text{-}T}+dE_{kin\text{-}T}$ is simply the density of electron states, D, multiplied by their probability of being occupied and by the differential element. Using the free electron model, the density of states is given by

$$D(E_{kin-T}) = \frac{8\sqrt{2}\pi (m^*)^{\frac{3}{2}}}{h^3}\sqrt{E_{kin-T}} \tag{S1}$$

The occupation of these states is governed by Fermi-Dirac statistics:

$$f(E_{kin-T}) = \frac{1}{1+\exp\left(\frac{E_{kin-T}-E_F}{kT}\right)} \tag{S2}$$

With the assumption of an isotropic effective mass, any $E_{kin\text{-}T}$ defines a sphere in velocity space. This represents all of the different velocity vectors whose magnitude corresponds to the chosen $E_{kin\text{-}T}$. If we also assume that there is no preferred direction for thermal motion, then all of the electrons with a chosen $E_{kin\text{-}T}$ would be evenly distributed over the surface of the corresponding sphere. The radius of the sphere is the thermal velocity, v_{th}.

The velocity normal to the interface ($v_{th\text{-}x}$) is a function of position on the surface of the sphere. If we choose a differential surface area element, then all of the electrons in that differential element will have the same $v_{th\text{-}x}$, and so the current for this element can be calculated. Integrating over half of the sphere (only those electrons traveling towards the interface) and over all energy levels, we get

$$j = q\int_0^\infty \int_0^{\frac{\pi}{2}} \frac{1}{2} v_{th-x} D(E_{kin-T}) f(E_{kin-T}) P(E_{kin-x}) \sin\theta \, d\theta dE_{kin-T} \tag{S3}$$

Using $v_{th-x} = v_{th}\cos\theta$, we can move the thermal velocity, density of states and Fermi-Dirac distribution terms outside of the θ integral, since they are all constants for a particular sphere:

$$j = \frac{q}{2}\int_0^\infty v_{th} D(E_{kin-T}) f(E_{kin-T}) \int_0^{\frac{\pi}{2}} P(E_{kin-x}) \sin\theta\cos\theta \, d\theta dE_{kin-T} \tag{S4}$$

Since $E_{kin\text{-}T}$ is a constant in the θ integral, we can convert it to an integral over $E_{kin\text{-}x}$ in the following manner:

$$E_{kin-x} = E_{kin-T}\cos^2\theta\,, \quad \therefore\ \frac{dE_{kin-x}}{d\theta} = -2E_{kin-T}\cos\theta\sin\theta \tag{S5}$$

Therefore,

$$j = \frac{q}{2}\int_0^\infty v_{th} D(E_{kin-T}) f(E_{kin-T}) \int_0^{E_{kin-T}} \frac{1}{2E_{kin-T}} P(E_{kin-x}) dE_{kin-x} dE_{kin-T} \tag{S6}$$

We will introduce a new term, $E_{kin\text{-}p}$, which is the kinetic energy associated with motion parallel to the interface plane such that $E_{kin\text{-}T} = E_{kin\text{-}x} + E_{kin\text{-}p}$. Changing the order of integration in Eq. (S6) and changing $E_{kin\text{-}T}$ to $E_{kin\text{-}p}$, we arrive at

$$j = \frac{q}{4}\int_0^\infty P(E_{kin-x}) \int_0^\infty \frac{1}{E_{kin-T}} v_{th} D(E_{kin-T}) f(E_{kin-T}) dE_{kin-p} dE_{kin-x} \tag{S7}$$

Substituting Eqs. (S1) and (S2) and using $v_{th} = \sqrt{\frac{2E_{kin-T}}{m^*}}$, we find that the $E_{kin\text{-}T}$ terms cancel:

$$j = \frac{4\pi q m^*}{h^3}\int_0^\infty P(E_{kin-x}) \int_0^\infty \frac{1}{1+\exp\left(\frac{E_{kin-x}+E_{kin-p}-E_F}{kT}\right)} dE_{kin-p} dE_{kin-x} \tag{S8}$$

If the $E_{kin\text{-}p}$ integral is performed explicitly, we arrive at Eq. (5) from the main text, and by comparing with Eq. (3) from the main text, we get Eq. (4) for the number of hits.
